# Discontinuing contact precautions for COVID-19: the science says its time

**DOI:** 10.1017/ice.2026.10482

**Published:** 2026-07

**Authors:** James M. Jurica, Davey M. Smith, Shira Abeles, Francesca J. Torriani, Daniel A. Sweeney

**Affiliations:** 1 Medicine/Pulmonary & Critical Care, University of California San Diego, La Jolla, USA; 2 Medicine/Infectious Disease, University of California San Diego, La Jolla, USA

## Abstract

The Centers for Disease Control and Prevention has recommended contact precautions for healthcare personnel caring for COVID-19 patients since the beginning of the pandemic. However, current scientific evidence points to transmission through small respiratory droplets or aerosols and not contaminated fomites as the dominant routes of transmission of SARS-CoV-2. We believe science shows there is no benefit and thus only negative consequences to patients, the environment, and the U.S healthcare system associated with ongoing contact precautions for patients with SARS-CoV-2 infection, and we advocate for updated guidelines reflecting current science.

## Introduction

The COVID-19 pandemic prompted a worldwide effort to understand the pathogenicity and transmission of a new virus at unprecedented speed. With limited data, the Centers for Disease Control and Prevention (CDC) provided guidance on how to minimize the transmission of infection. Consistent with previous outbreaks of high consequence and novel respiratory viruses, the CDC wisely recommended a comprehensive strategy including usage of an N95 respirator, eye protection, and contact precautions (gowns and gloves) to prevent the transmission of SARS-CoV-2 in the healthcare setting at the beginning of the COVID-19 pandemic before the routes of transmission had been fully elucidated. Three years after the end of the pandemic was declared, we posit that there is ample data to remove contact precautions from national and state guidelines. In this commentary, we explore the historical and scientific basis for the initial COVID-19 contact precaution recommendation, subsequent data emphasizing respiratory droplet and aerosol transmission, the consequences of the ongoing use of contact precautions, and propose a streamlined guide to safely care for patients with COVID-19.

## Historical and scientific basis for instituting contact precautions for patients with proven or suspected SARS-CoV-2 infection at onset of the COVID-19 pandemic

Early in the pandemic, the method of transmission of SARS-CoV-2 was unclear, with early research suggesting that fomites could be a significant source of transmission.^1–3^ For this reason, the CDC recommended in March 2020 that healthcare personnel (HCP) treating patients with suspected or proven COVID-19 adhere to both modified airborne and contact precautions, i.e., an N95 respirator, gown, gloves, and eye protection.^4,5^ By May of 2020, however, the CDC determined fomites to be an unlikely route of transmission, but difficulty in completely ruling out fomite transmission led to an abundance of caution. Six years later, this CDC infection-control recommendation driving healthcare policy for patients with suspected or proven COVID-19 remains in effect and is widely followed by US hospitals.^6^

## Subsequent data supports airborne and droplet but not fomite transmission of SARS-CoV-2

Understanding of the transmission of SARS-CoV-2 has evolved. For example, clinical studies evaluating “superspreader” events have provided compelling evidence for airborne and droplet transmission through identifications of patterns of transmission based on spatial clustering in poorly ventilated environments.^7,8^ Similarly, quantitative modeling analysis of these same events has demonstrated that aerosols are the main route of transmission, with the risk of infection via fomite estimated to be less than 1 in 10,000.^9,10^ Likewise, animal studies evaluating transmission of SARS-CoV-2 have consistently demonstrated that while transmission via fomite is theoretically possible, airborne and droplet are by far the dominant routes of transmission and result in more severe disease.^11,12^

## The consequences of ongoing contact precautions for patients with COVID-19

Given negligible fomite transmission, contact precautions are not protecting HCP or patients from contracting COVID-19; rather, contact precautions are having detrimental effects. Studies of patients on contact precautions for multidrug-resistant organisms (MDRO) report lower staff responsiveness (63% vs 51%) and more problems with inpatient care. Patients on contact precautions are three times less likely to be satisfied with healthcare worker assistance compared with patients not on contact precautions.^13–15^ They also have fewer visitors when compared to patients not on contact precautions and have higher in-hospital anxiety scores.^15,16^ Additionally, HCP are less likely to interact with patients on contact precautions (2.78 vs 4.37 visits per hour).^16^ This may be, at least in part, due to the time needed to don and doff gowns, which was estimated to take 45,277 hours/year for two California hospitals according to a 2016 study by Martin et al.^17^ These observations have contributed to the rationale for many US healthcare institutions to stop requiring contact precautions for highly endemic methicillin-resistant *Staphylococcus aureus* or vancomycin-resistant *Enterococcus*.^18^

These detrimental effects are not limited to the inpatient setting. The requirement to don and doff personal protective equipment (PPE) for confirmed or suspected COVID-19 has been estimated to add 3.9 minutes per outpatient encounter.^19^ In a recent survey of HCP practicing at walk-in clinics, 54% reported that isolation precautions increased burden on HCP, 69% reported increased time required per patient encounter due to donning and doffing of PPE, and 46% reported that PPE increased difficulty associated with collection and management of specimens.^20^

**Figure 1.**
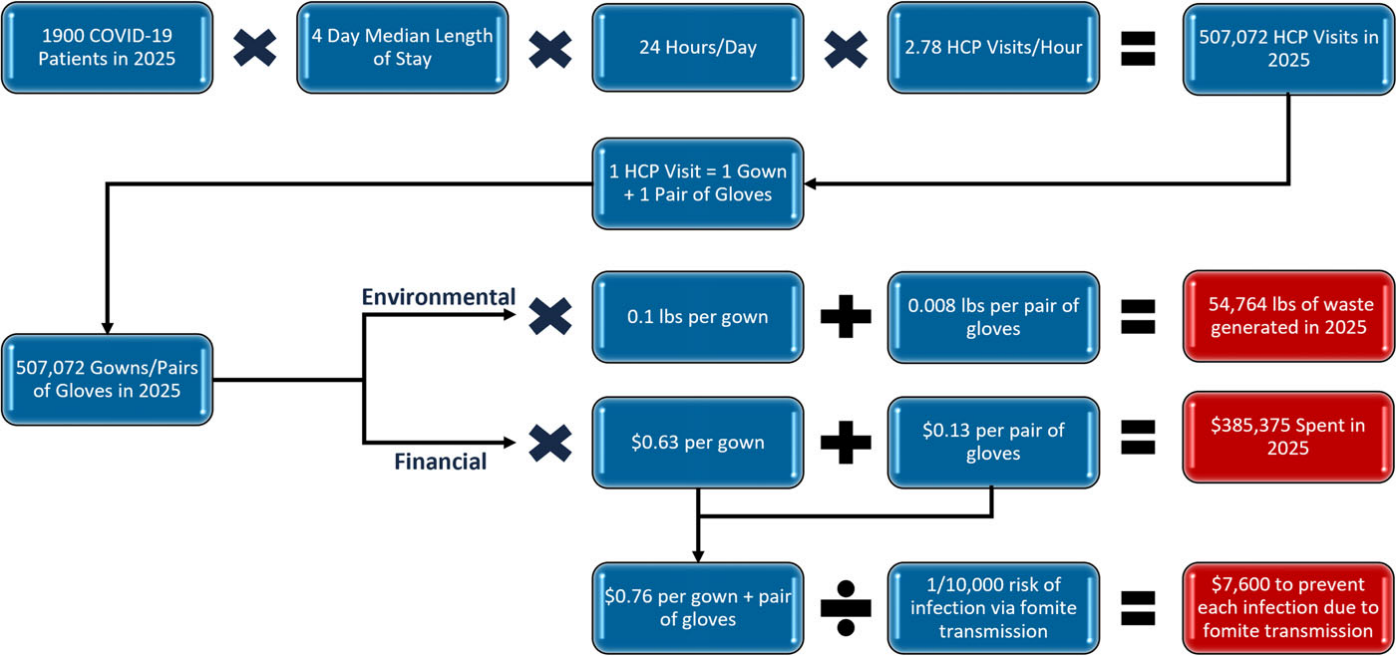
Figure 1 long description. Financial & environmental impact of COVID-19 contact precautions at UC San Diego Health in 2025.

There are also financial and environmental impacts of contact precautions for patients with suspected or proven COVID-19. Based on data for our academic institution, we estimate the added financial cost of utilizing gowns and gloves to care for 1900 patients admitted with COVID-19 in 2025 was $385,375 (Figure 1).^21,22^ To assess the environmental cost, we estimated the waste produced and calculated that this practice generated 54,764 pounds of discarded gloves and gowns at our institution in 2025.^23,24^ These figures reflect the burden at a single hospital system; when extrapolated across the entire healthcare system the financial and environmental cost of contact precautions for patient with suspected or proven COVID-19 would be magnified many fold. Furthermore, this policy is not cost-efficient. We estimate $7,600 would need to be spent on gowns and gloves to prevent a single case of COVID-19 transmitted via fomite. Admittedly, these calculations rely heavily on generalized assumptions and lack nuance; nonetheless, because this analysis does not account for usage of gowns and gloves by visitors of patients with COVID-19, costs of waste disposal, or usage of gowns and gloves in the outpatient setting, these results certainly underestimate the true cost of this policy.

## Proposed future steps

Discontinuation of the routine use of contact precautions for patients with SARS-CoV-2 is unlikely to be considered a radical policy change in 2026, with experts having called for reconsideration of this practice since 2023.^25,26^ Many countries (e.g. Australia, The European Union, and Singapore) and some U.S. states have already relaxed contact precautions; both the Massachusetts and Washington Departments of Health now recommend the routine use of gowns and gloves only when contact with potentially infectious material is anticipated.^26–29^ While subsequent data from this policy change are limited, a single retrospective study from Singapore showed discontinuation of contact precautions resulted in significant cost and environmental savings with no increase in infection rate among HCP or patients.^30^ A similar outcome would be anticipated in the United States, where CDC surveillance and data analytics demonstrate substantially decreased test positivity, emergency room visits due to COVID-19, hospitalization rates, and deaths due to COVID-19 in 2026 when compared to rates from the peak of the pandemic.^31^ Collectively, these data suggest that SARS-CoV-2 infection is currently associated with lower transmission and clinical severity, further reducing the likelihood that relaxation of contact precautions would result in increased nosocomial transmission and harm. Such a policy change would align with influenza infection control practices. Notably, viable influenza virus can be recovered from environmental surfaces at low levels, whereas no published studies have demonstrated recovery of viable SARS-CoV-2 from fomites.^32,33^

We recommend the thoughtful discontinuation of contact precautions for COVID-19. At a health-system level, prospective monitoring of hospital-onset COVID-19 should be continued. At county and state agencies, systems should collect data and analyze and respond if hospital-onset clusters of SARS-CoV2 infection rates among HCP or patients occur without an increase in the prevalence in the community.

In summary, CDC guidance at the start of the pandemic was thoughtful and appropriately cautious in the face of a novel and lethal respiratory pathogen. However, our understanding of SARS-CoV-2 transmission has evolved since the beginning of the pandemic. For the sake of the environment, the financial viability of our healthcare system, and our HCPs, patients, and visitors, we should no longer mandate contact precautions for patients with suspected or confirmed COVID-19. The science says it’s time.

## Figure 1. Long description

There were 1900 patients hospitalized with COVID-19, each with a median length of stay of 4 days and an average of 2.78 healthcare personnel (HCP) visits per hour, corresponding to 507,072 HCP visits annually. Each visit required one gown and one pair of gloves. At 0.1 lb per gown and 0.008 lb per pair of gloves, this resulted in an estimated 54,764 lb of waste. The per-unit costs are $0.63 per gown and $0.13 per pair of gloves, totaling $385,375 annually. The combined cost per patient⏧HCP interaction is $0.76. Assuming a fomite transmission risk of 1 in 10,000 per encounter, the estimated cost to prevent one infection via fomite transmission is $7,600.

Navigate back to Figure 1..
